# Model of Acute Liver Failure in an Isolated Perfused Porcine Liver—Challenges and Lessons Learned

**DOI:** 10.3390/biomedicines10102496

**Published:** 2022-10-06

**Authors:** Joshua Hefler, Sanaz Hatami, Aducio Thiesen, Carly Olafson, Kiarra Durand, Jason Acker, Constantine J. Karvellas, David L. Bigam, Darren H. Freed, Andrew Mark James Shapiro

**Affiliations:** 1 Division of General Surgery, Department of Surgery, Faculty of Medicine & Dentistry, University of Alberta, Edmonton, AB T6G 2R3, Canada; 2 Department of Medicine, Faculty of Medicine & Dentistry, University of Alberta, Edmonton, AB T6G 2R3, Canada; 3 Canadian Donation & Transplantation Research Program, Edmonton, AB T6G 2R3, Canada; 4 Department of Laboratory Medicine & Pathology, Faculty of Medicine & Dentistry, University of Alberta, Edmonton, AB T6G 2R3, Canada; 5 Canadian Blood Services, Edmonton, AB T6G 2R3, Canada; 6 Division of Gastroenterology, Department of Medicine, Faculty of Medicine & Dentistry, University of Alberta, Edmonton, AB T6G 2R3, Canada; 7 Department of Critical Care Medicine, Faculty of Medicine & Dentistry, University of Alberta, Edmonton, AB T6G 2R3, Canada; 8 Division of Cardiac Surgery, Department of Surgery, Faculty of Medicine & Dentistry, University of Alberta, Edmonton, AB T6G 2R3, Canada; 9 Clinical Islet Transplant Program, University of Alberta, Edmonton, AB T6G 2R3, Canada

**Keywords:** acute liver failure, porcine model, ex situ machine perfusion, acetaminophen, carbon tetrachloride

## Abstract

Acute liver failure (ALF) is a rare but devastating disease associated with substantial morbidity and a mortality rate of almost 45%. Medical treatments, apart from supportive care, are limited and liver transplantation may be the only rescue option. Large animal models, which most closely represent human disease, can be logistically and technically cumbersome, expensive and pose ethical challenges. The development of isolated organ perfusion technologies, originally intended for preservation before transplantation, offers a new platform for experimental models of liver disease, such as ALF. In this study, female domestic swine underwent hepatectomy, followed by perfusion of the isolated liver on a normothermic machine perfusion device. Five control livers were perfused for 24 h at 37 °C, while receiving supplemental oxygen and nutrition. Six livers received toxic doses of acetaminophen given over 12 h, titrated to methemoglobin levels. Perfusate was sampled every 4 h for measurement of biochemical markers of injury (e.g., aspartate aminotransferase [AST], alanine aminotransferase [ALT]). Liver biopsies were taken at the beginning, middle, and end of perfusion for histological assessment. Acetaminophen-treated livers received a median dose of 8.93 g (8.21–9.75 g) of acetaminophen, achieving a peak acetaminophen level of 3780 µmol/L (3189–3913 µmol/L). Peak values of ALT (76 vs. 105 U/L; *p* = 0.429) and AST (3576 vs. 4712 U/L; *p* = 0.429) were not significantly different between groups. However, by the end of perfusion, histology scores were significantly worse in the acetaminophen treated group (*p* = 0.016). All acetaminophen treated livers developed significant methemoglobinemia, with a peak methemoglobin level of 19.3%, compared to 2.0% for control livers (*p* = 0.004). The development of a model of ALF in the ex vivo setting was confounded by the development of toxic methemoglobinemia. Further attempts using alternative agents or dosing strategies may be warranted to explore this setting as a model of liver disease.

## 1. Introduction

Acetaminophen toxicity is the most common cause of acute liver failure (ALF) in North America [1]. Despite its low occurrence (10 people per million annually), it is associated with high morbidity and a devastating mortality rate of almost 45% [2]. Early presentations (within 24 h) may respond to treatment with N-acetylcysteine (NAC), which replenishes the liver’s glutathione reserve and facilitates the conversion of acetaminophen into non-toxic metabolites [3,4]. NAC can delay progression, but, otherwise, management is largely supportive, allowing the liver time to recover. Liver transplantation may be considered based on the severity of injury, as judged by the King’s College criteria, and the patient’s pre-morbid status [5]. While survival rate is high after transplantation (75% at 5 years), transplantation itself is associated with its own morbidity and requires lifelong immunosuppression, which is not to mention the limited supply of suitable organs [6,7].

Acetaminophen is a common over-the-counter analgesic. It is primarily metabolized in the liver, where it undergoes either glucuronidation or sulfonation by cytochrome P450 enzymes to generate water soluble metabolites that can be readily excreted in the urine [8]. A small proportion is oxidized into N-acetyl-p-benzoquinone (NAPQI). NAPQI is a strong oxidizing compound that generates oxidative stress and causes cellular dysfunction by forming acetaminophen-protein adducts [9]. NAPQI can be further metabolized into non-toxic compound by glutathione dependent pathways [10]. However, when the main pathways of acetaminophen metabolism become saturated and glutathione stores are depleted, accumulation of NAPQI can lead to hepatocyte death and severe liver injury [9].

Pre-clinical models used to study acetaminophen toxicity in the liver vary from hepatocytes cultured in vitro, to small (e.g., rodents) and large animal (e.g., dogs, primates, pigs) models. More advanced models, including use of bio-artificial liver and 3D cell culture, have also been employed [10,11,12]. Porcine models are typically preferred as large animal models, due to their similarity to humans in terms of liver anatomy, histology and metabolism, as well as logistic considerations (e.g., size, handling, availability) [13,14]. Previous methods to induce ALF with acetaminophen in pigs have required the animals to be maintained under anesthesia for the duration of the experiment (typically > 24 h), which are expensive, technically challenging, and confounded by ethical considerations [15,16].

Ex vivo normothermic machine perfusion (NMP) may provide an alternative approach and more ethically acceptable platform for modeling ALF by isolating the injury to the target organ rather than the entire animal. NMP is a technology that aims to maintain normal liver physiology and metabolism outside of the body [17]. It was developed with the intent of preserving and assessing grafts before transplantation, such as marginal grafts donated after cardiovascular death. Its clinical utility was demonstrated in a randomized controlled trial published in 2018 by Nasralla and colleagues, where minimal liver injury was observed in grafts subjected to NMP, despite lower discard rate and longer preservation time, when compared to traditional cold static storage [18]. Related preclinical research has focused on graft treatment while on the ex vivo circuit, including ‘de-fatting’ steatotic livers, gene therapy, and eradication of hepatitis C [19,20,21,22] Several other exciting potential applications of NMP outside of transplantation, such as use in toxicology studies, cancer research, and liver support remain to be explored [23].

Development of reproducible, translatable ALF models are needed to facilitate the search for novel treatments. An ALF model in an isolated organ has several potential advantages over in vivo models, including improving animal welfare and extending treatment duration. However, the impact that the lack of other, potentially compensatory mechanisms, provided by other organ systems will have is unknown, as are the effects of interaction with artificial circuit components. Herein, we describe our approach to creating a large animal ex vivo model of ALF using acetaminophen, as well as another common agent, carbon tetrachloride, and describe the limitations we encountered.

## 2. Materials and Methods

This study used domestic swine, 3–4 months of age, weighing 40–50 kg, at which point their livers are similar in size to an adult human (~1200 g), while still being easily manageable in our surgical facility. Only female animals were used as male animals are routinely castrated to prevent aggression and boar taint (if used for meat), which can lead to visceral fat accumulation, among other physiological changes. Animals were supplied by the Swine Research and Technology Centre at the University of Alberta. Animals were obtained from different litters, randomly housed, and allocated to treatment groups. This study was conducted in accordance with the Canadian Council on Animal Care Guidelines and Policies with approval from the Animal Care and Use Committee (Health Sciences) for the University of Alberta (AUP00001036; approved 04/07/2014), which ensure the ethical treatment of research animals.

### 2.1. Liver Procurement

Hepatectomy was performed in fasted, anesthetized pigs, as in our previously established protocol [24,25]. In brief, animals were premedicated with 20 mg/kg of ketamine (Ketaset; Zoetis Canada Inc., Kirkland, QC, Canada) and 0.05 mg/kg of atropine (Atro-SA; Rafter 8 Products Inc., Calgary, AB, Canada), followed by endotracheal intubation using direct laryngoscopy. During the procedure, animals were maintained on inhalational anesthesia (3–4% isoflurane; Frensenius Kabi Canada, Toronto, ON, Canada). An orogastric tube was also inserted to decompress the stomach.

Animals were prepped with a 7.5% povidone-iodine solution and draped appropriately. Surgery was conducted under aseptic technique, including the use of sterilized instruments. An 8 Fr venous cannula was inserted into the right internal jugular vein for fluid administration. A midline laparotomy was performed to expose the intra-abdominal contents. The bowel was displaced from the abdomen to expose to the infra-renal aorta, which was then dissected and encircled with 0-silk ties (Perma Hand; Ethicon Inc., Bridgewater, NJ, USA) for ease of future cannulation. The bowel was returned to abdomen and rolled laparotomy sponges were place beneath the liver to elevate it into the surgical field. The fundus of the gallbladder was grasped with an Allis clamp and retracted superiorly to facilitate dissection from the gallbladder fossa. The cystic duct and artery were then ligated and the gallbladder was disposed of. The porta hepatis was exposed and the common bile duct was identified and ligated just prior to it entering the pancreas. The main portal vein was then dissected free of its peritoneal covering and tagged with a silk tie. A portion of the upper intra-abdominal aorta was also dissected and tagged with a silk tie for subsequent clamp placement during abdominal aortic flush.

Blood to be used in the perfusion was collected via the right atrium. To facilitate this, a midline sternotomy was performed and the heart was exposed and freed from the pericardium and associated tissues. The superior portion of the right atrium was encircled with a 4-0 polypropylene suture (Prolene Blu; Ethicon Inc., Bridgewater, NJ, USA). An incision was made in the atrium above the suture and a 28 French two-stage venous cannula was inserted and secured with a vascular torniquet. Blood was collected into a sterile container to the point of complete exsanguination (1.5–2 L).

Upon completion of exsanguination, an aortic cross-clamp was placed on the upper portion of the abdominal aorta previously identified to prevent flow of the preservation solution into the chest. The infra-renal aorta was then cannulated and 2 L of cold (4 °C) histidine-tryptophan-ketoglutarate solution (Custodiol^®^ HTK; Essential Pharmaceuticals LLC., Durham, NC, USA) was used to flush the intra-abdominal organs. Ice was placed into the abdomen to cool the organs and the inferior vena cava above the diaphragm was incised to vent the flush solution. Following completion of the intra-abdominal flush, the liver was resected and weighed on the back table.

### 2.2. Normothermic Machine Perfusion (NMP)

Livers were perfused according to our previously established protocol [24,25]. A schematic of our NMP circuit is provided in Supplementary Figure S1. Our custom designed circuit consisted of an EOS ECMO oxygenator (Sorin Group Canada Inc., Burnaby, BC, Canada), a MYOtherm XP heat exchanger (Medtronic of Canada Ltd., Brampton, ON, Canada), and two BPX-80 Bi-Medicus centrifugal pumps (Medtronic of Canada Ltd., Brampton, ON, Canada). Perfusate draining from the liver was collected in a filtered CARD EVO cardiotomy reservoir (Sorin Group Canada Inc., Burnaby, BC, Canada) before recirculation through the pumps and was heated using a CW-05G water bath (Lab Companion, Jeio Tech Inc., Billerica, MA, USA). Hepatic artery and portal venous flows were measured using Transonic Clamp-on Tubing Flowsensors (6PXL and 7PXL; Transonic Systems Inc., Ithaca, NY, USA) and pressures were measured using TruWave pressure transducers (Edwards Life Sciences Canada Inc., Mississauga, ON, Canada). The centrifugal pumps were computer-controlled to maintain desired hepatic artery pressure and portal venous flow. Inflows of oxygen and carbon dioxide were titrated to maintain a partial pressure of arterial oxygen between 100 and 120 mmHg and a partial pressure of carbon dioxide between 35 and 45 mmHg.

The circuit was primed with a 1:1 ratio of whole blood to modified Krebs-Henseleit solution (glucose [5 mM], sodium chloride [85 mM], potassium chloride [5 mM], calcium chloride [1 mM], magnesium chloride [1 mM], sodium bicarbonate [25 mM], sodium phosphate monobasic [1 mM], sodium pyruvate [5 mM], and 8% bovine serum albumin). Piperacillin/tazobactam (3.375 g; Sandoz Canada, Boucherville, QC, Canada), methylprednisolone (500 mg; Solu-Medrol, Pfizer Canada Inc., Kirkland, QC, Canada), and sodium heparin (5000 U; Fresenius Kabi Canada, Toronto, ON, Canada) were added prior to attaching the organ. During perfusion, the organ received infusions of regular insulin (2 U/h; Humulin R; Eli Lilly & Company, Toronto, ON, Canada) and sodium heparin (1000 U/h). Piperacillin/tazobactam and methylprednisolone were re-dosed after 12 h. Perfusate pH and glucose was maintained within physiological range (7.35–7.45 and 6–10 mmol/L).

Prior to attaching the liver to the circuit, it was flushed with 2 L of normal saline. The hepatic artery and portal vein were canulated with an 8 Fr arterial cannula (Medtronic of Canada Ltd., Brampton, ON, Canada) and 1/4’ polycarbonate tubing connector, respectively. The common bile duct was cannulated with tubing that drained to a reservoir outside of the organ chamber. The perfusate was heated to maintain the organ at 37 °C. Arterial and portal venous flows were gradually increased to physiological values, as the organ warmed.

### 2.3. Experimental Protocol

Control livers were run for 24 h (*n* = 5). Acetaminophen-treated livers (*n* = 6) received doses of acetaminophen (Sigma-Aldrich Canada, Oakville, ON, Canada) dissolved directly into the perfusate, starting 1 h after beginning perfusion. Acetaminophen was dosed at 30-min intervals up to 12 h, with the dose titrated to avoid excessive methemoglobinemia. Acetaminophen-treated livers were intended to run for 24 h, but were stopped early as necessitated by perfusion parameters and circuit volume. These livers similarly received additives necessary to maintain physiological blood gas parameters. Since we were unable to achieve sufficient toxicity without causing methemoglobinemia, further perfusions were discontinued, which lead to unequal numbers between groups. An additional experimental group treated with carbon tetrachloride (CCl_4_; Sigma-Aldrich Canada, Oakville, ON, Canada) as the hepatotoxic agent was also attempted, but discontinued after two perfusions, due to circuit and perfusate incompatibility.

### 2.4. Perfusate Biochemistry

Perfusate was sampled from the hepatic artery and portal venous inflows and vena cava outflow every four hours and as needed to measure hemoglobin, methemoglobin, pH, electrolytes, lactate, glucose, and partial pressures of oxygen and carbon dioxide using a point-of-care blood gas machine (ABL Flex Analyzer; Radiometer Canada, Mississauga, ON, Canada). Perfusate sampled from the vena cava outflow was also measured for alanine aminotransferase (ALT), aspartate aminotransferase (AST), lactate dehydrogenase (LDH), and total and conjugated bilirubin using a Beckman Coulter Unicel Dxc800 Syncron (Beckman Coulter Canada LP, Mississauga, ON, Canada).

### 2.5. Histology

Incisional liver biopsies were taken at 2, 12 and 24 h into perfusion from the right lateral lobe, a portion of which was fixed in 10% formalin. These samples were then embedded in paraffin, stained with hematoxylin and eosin, and examined in a blinded fashion by an expert pathologist (AT). Samples were rated on the presence of necrosis, vacuolization, and congestion, as previously reported [26].

### 2.6. Chromogenic and Enzyme-Linked Immunosorbent Assays

Both malondialdehyde (MDA) and glutathione were measured in tissue using colorimetric assay kits (ab118970, Abcam PLC, Cambridge, UK; Invitrogen EIAGSHC, Fisher Scientific Co., Edmonton, AB, Canada), run according to manufacturer’s instructions. Samples and standards were run in duplicate to a 96-well microplate and absorbance was read on a spectrophotometer (Multiskan SkyHigh; Fisher Scientific Co., Edmonton, AB, Canada) measuring optical density at 532 and 405 nm, respectively. Oxidized low-density lipoprotein (oxLDL) and haptoglobin were measured in perfusate samples using porcine-specific enzyme-linked immunosorbent assays (ELISA) (MBS2508909, MyBioSource Inc., San Diego, CA, USA; ab205091; Abcam PLC, Cambridge, UK), run according to manufacturer’s instructions.

For determination of free hemoglobin (an indicator of hemolysis), perfusate samples were centrifuged (2200× *g*, 10 min, 4 °C) and the supernatant in was diluted in Drabkin’s reagent (0.61 mmol/L potassium ferricyanide, 0.77 mmol/L potassium cyanide, 1.03 mmol/L potassium dihydrogen phosphate, and 0.1% [*vol/vol*] TritonX-100 [Sigma-Aldrich Canada, Oakville, ON, Canada]). Trilevel Hb controls (StanBio Laboratory, Boerne, TX, USA) were used for quality control. Absorbances were read on a spectrophotometer (SpectraMax 384 Plus, Molecular Devices Corp., Sunnyvale, CA, USA) at 540 nm and free hemoglobin concentration was determined using the calculation previously described in the literature [27].

### 2.7. In Vitro Testing of Acetaminophen Metabolites and CCl_4_

The effect of acetaminophen metabolites on methemoglobinemia was tested in vitro by adding them to a sample of perfusate (1:1 whole blood with modified Krebs-Henseleit). P-aminophenol (Sigma Aldrich Canada, Oakville, ON, Canada) was added in a concentration of 100 µg/mL and let sit at room temperature. Methemoglobin was measured, as described above, at 30 and 90 min. N-acetyl-p-benzoquinone imine (NAPQI) (Sigma Aldrich Canada, Oakville, ON, Canada) was added to a separate sample of perfusate at a concentration of 125 µg/mL and methemoglobin was again measured after 90 and 180 min at room temperature. For comparison, acetaminophen (Sigma Aldrich Canada, Oakville, ON, Canada) was added to perfusate at a concentration of 10 mg/mL and methemoglobin was measured after 60 min. The effect of CCl_4_ on hemolysis was tested in vitro by adding CCl_4_ (Sigma Aldrich Canada, Oakville, ON, Canada) to whole blood in a concentration of 10 µL/mL and measuring plasma free hemoglobin after 30 min at room temperature, as detailed above. Free hemoglobin was measured in porcine whole blood after 30 min at room temperature without the addition of CCl_4_ for control comparison.

### 2.8. Statistical Analysis

Data are reported as medians (interquartile range). The Mann U Whitney test was used to compare single measures between the two groups, as well as repeated measures at different time points. A *p*-value of <0.05 was considered significant. All analyses were performed using GraphPad Prism v9 (GraphPad Software Inc., San Diego, CA, USA).

## 3. Results

### 3.1. Perfusion Parameters

All control livers were perfused for 24 h (*n* = 5), while acetaminophen-treated livers (*n* = 6) we perfused for a median time of 892 (566–1316) minutes. Peak hepatic artery flow and pressure were 480.1 mL/min and 59.9 mmHg in the untreated group and 565.3 mL/min and 50.1 mmHg in the acetaminophen treated group (*p* = 0.931 and 0.046, respectively) (Supplementary Figure S2). Peak portal venous flow and pressure were 902.1 mL/min and 7.4 mmHg in the untreated group and 759.2 mL/min and 8.2 mmHg in the acetaminophen treated group (*p* = 0.017 and 0.662, respectively). Median hourly bile production was no different between groups (7.7 mL/h vs. 7.0 mL/h; *p* = 0.610).

### 3.2. Perfusate Biochemistry

**Figure 1:**
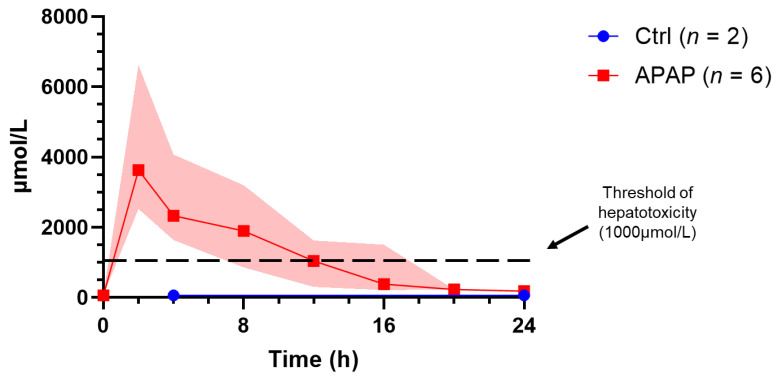
Acetaminophen (n-acetyl-para-aminophenol, APAP) concentration in perfusate over the duration of liver perfusion compared to control (Ctrl) livers without addition of APAP. The highlighted area represents the IQR.

**Figure 2:**
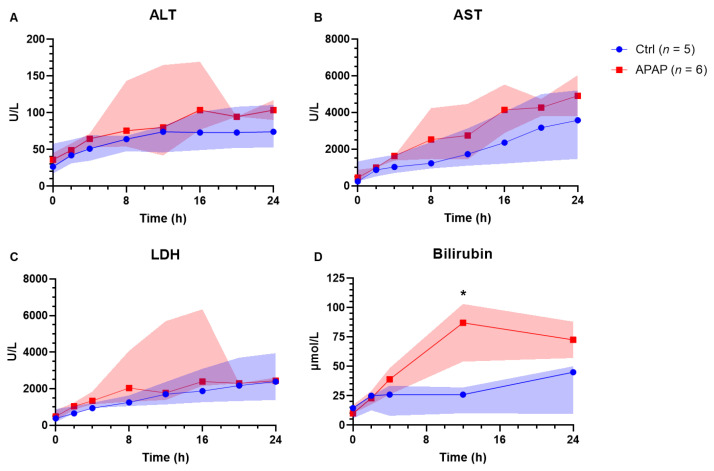
Perfusate concentrations of (**A**) alanine transaminase (ALT), (**B**) aspartate transaminase (AST), (**C**) lactate dehydrogenase (LDH), and (**D**) total bilirubin over the duration of perfusion in livers treated with acetaminophen (n-acetyl-para-aminophenol, APAP) compared to those without (Ctrl). The highlighted area represents IQR. * = *p* < 0.05.

**Figure 3:**
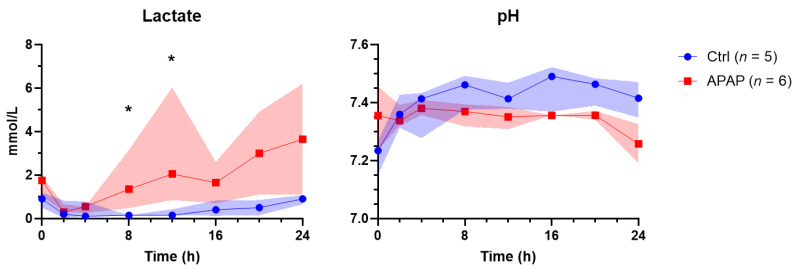
Perfusate (**A**) lactate and (**B**) pH over the duration of perfusion in livers treated with acetaminophen (n-acetyl-para-aminophenol, APAP) compared to those without (Ctrl). The highlighted area represents IQR. * = *p* < 0.05.

Acetaminophen-treated livers received a median dose of 8.93 g (8.21–9.75 g) of acetaminophen, achieving a median peak acetaminophen level of 3780 µmol/L (3189–3913 µmol/L). The acetaminophen concentration remained above the clinical threshold for toxicity (1000 µmol/L) up to 12 h of perfusion (Figure 1). Peak values of ALT (76 vs. 105 U/L; *p* = 0.429), AST (3576 vs. 4712 U/L; *p* = 0.429), and LDH (2389 vs. 2496 U/L; *p* = 0.537) were not significantly different between groups (Figure 2). There was a trend toward peak bilirubin being higher in the acetaminophen-treated group (45 vs. 57 µmol/L; *p* = 0.056). End lactate was significantly higher (1.0 vs. 4.4 mmol/L; *p* = 0.011) and end pH was significantly lower (7.42 vs. 7.31; *p* = 0.030) in the acetaminophen-treated group, despite using more THAM for pH correction (33.0 vs. 55.5 mL; *p* = 0.004) (Figure 3). There was also a significant difference in perfusate INR, which, over the course of the perfusion, fell by 1.65 points (to 1.65 [1.53–2.45]) in the control group and rose by 0.45 points (to 4.0 [3.93–4.45]) in the acetaminophen-treated group (*p* = 0.029).

### 3.3. Histology

**Figure 4:**
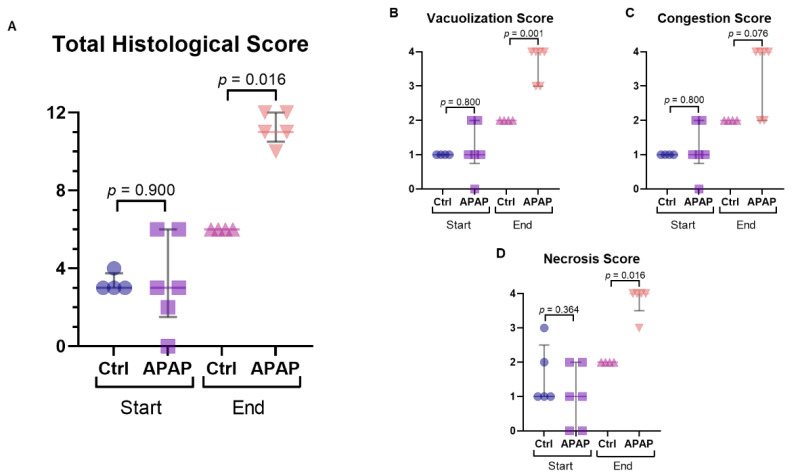

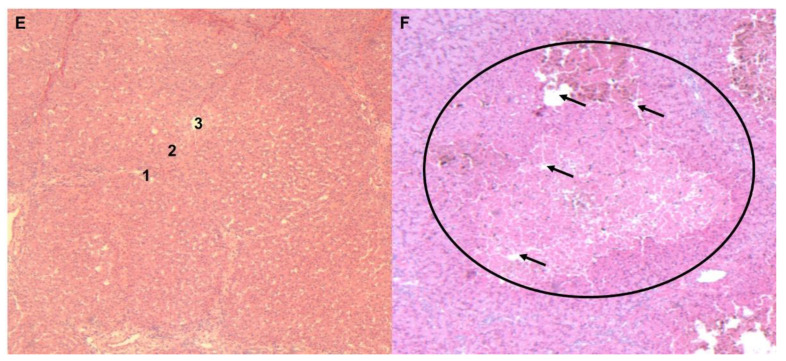
Histological scoring for control (Ctrl) and acetaminophen-treated (n-acetyl-para-aminophenol, APAP) livers. Comparisons made at the start and end of perfusion based on (**A**) total histological score and sub-scores of (**B**) vacuolization, (**C**) congestion, and (**D**) necrosis. The data is presented as median and IQR. A representative image showing preserved hepatic architecture is shown (**E**), with zones 1–3 readily identifiable. This is contrasted with an image from an acetaminophen treated liver (**F**) showing pan-lobular hepatocyte necrosis and congestion (circle) and diffuse sinusoidal dilation (arrows).

Histological scoring was similar between groups at the start of perfusion (*p* = 0.900). However, by the end was significantly worse in the acetaminophen treated group (*p* = 0.016) (Figure 4). Specifically, sub-scores of necrosis and vacuolization were significantly higher in the treated group by the end of perfusion (*p* = 0.016 and 0.001, respectively).

### 3.4. Measures of Oxidative Stress

**Figure 5:**
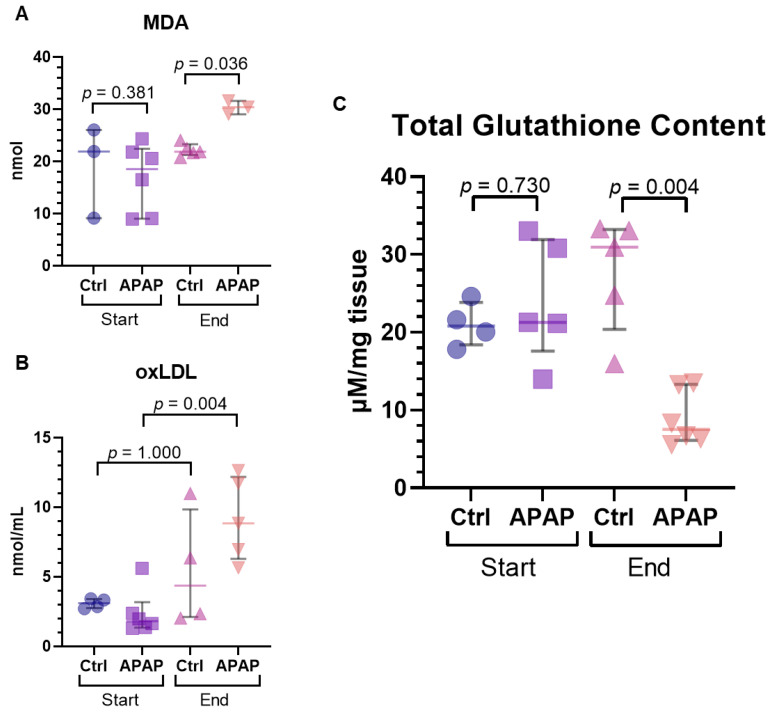
Markers of oxidative stress during liver perfusion in livers treated with acetaminophen (n-acetyl-para-aminophenol, APAP) compared to those without (Ctrl): (**A**) tissue malondialdehyde (MDA), (**B**) perfusate oxidized low-density lipoprotein (oxLDL), and (**C**) tissue glutathione. The data is presented as median and IQR.

Tissue MDA as a marker of oxidative stress was significantly increased in the acetaminophen-treated group at the end of perfusion compared to controls (21.9 [21.7–22.5] vs. 30.4 [29.1–31.6] nmol; *p* = 0.036) (Figure 5A). Perfusate oxLDL, a circulating marker of oxidative stress, was also found to be increased in the acetaminophen-treated group at the end compared to the start (1.81 [1.43–2.27] vs. 8.84 [6.95–11.72] nmol/mL; *p* = 0.004), though was not significantly different from end concentration of the controls (4.36 [2.27–7.52] nmol/mL; *p* = 0.191) (Figure 5B). As well, tissue glutathione content, which buffers against oxidative stress, was correspondingly decreased in acetaminophen-treated livers at the end of perfusion (31.0 [24.8–33.1] vs. 7.51 [6.4–12.0] µM/mg tissue; *p* = 0.004) (Figure 5C).

### 3.5. Methemoglobinemia and Hemolysis

**Figure 6:**
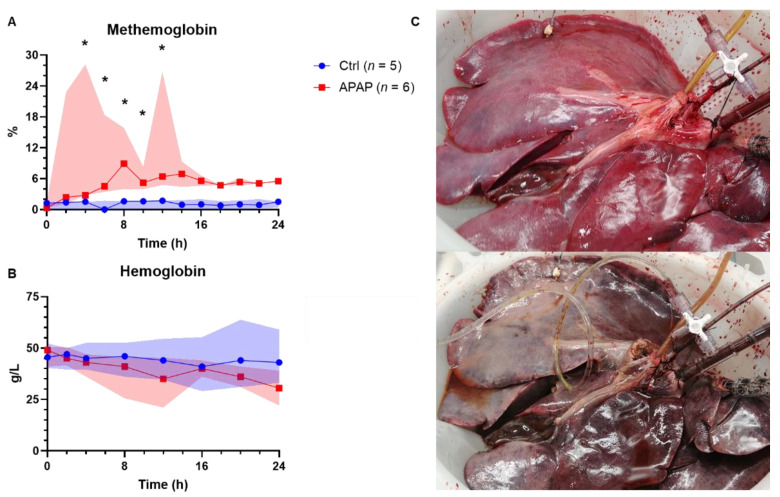
Effect of acetaminophen of perfusate (**A**) methemoglobin and (**B**) hemoglobin concentrations in livers treated with acetaminophen (n-acetyl-para-aminophenol, APAP) compared to those without (Ctrl). The highlighted area represents IQR; * = *p* < 0.05. Perfused liver before (top) and after (bottom) development of methemoglobinemia (**C**).

**Figure 7:**
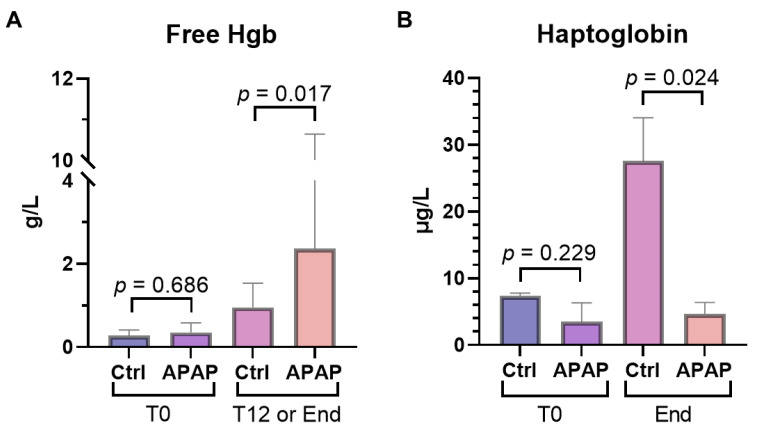
Perfusate (**A**) free hemoglobin (Hgb) and (**B**) haptoglobin over the course of liver normothermic perfusion in livers treated with acetaminophen (n-acetyl-para-aminophenol, APAP) compared to those without (Ctrl). The data is presented as median and IQR.

Methemoglobinemia, a well described complication of acetaminophen toxicity in in vivo animal models, was also observed in our ex vivo model (Figure 6) [28]. The median peak methemoglobulin level was 19.3% for acetaminophen-treated livers, compared to 2.0% for control livers (*p* = 0.004). Free hemoglobin, a measure of hemolysis, was significantly lower in controls at the midpoint of perfusion (12 h) compared to similar time points in acetaminophen-treated livers (0.96 [0.72–1.43] vs. 2.37 [2.10–7.65] g/L; *p* = 0.017), though was not significantly different at the end of perfusion (1.41 [1.08-3.02] vs. 2.15 [1.74–7.60] g/L; *p* = 0.247) (Figure 7A). Hemolysis was further evident in the median drop in hemoglobin over the course of the perfusion, which was 19.0 g/L for the acetaminophen-treated group and only 1.0 g/L for the control livers (*p* = 0.008). Median perfusate haptoglobin at the endpoint was significantly lower in the acetaminophen-treated group (27.56 [22.73–30.80] vs. 4.67 [2.90–6.09] µg/mL; *p* = 0.024) (Figure 7B).

**Figure 8:**
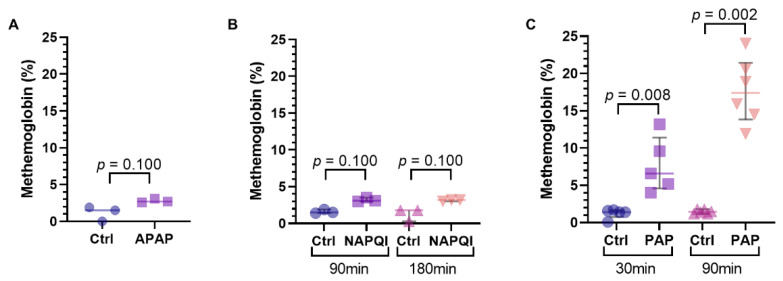
Effects of (**A**) acetaminophen and its metabolites, (**B**) n-acetyl-p-benzoquinone imine (NAPQI), and (**C**) p-aminophenol (PAP) on the development of methemoglobinemia in vitro. The data is presented as median and IQR.

It had been suggested that the metabolite of acetaminophen responsible for methemoglobinemia was PAP [29]. The addition 100 µg/mL of PAP in vitro to a sample of perfusate resulted in significant methemoglobin compared to the control after 30 (1.4% [0.8–1.6%] vs. 6.6% [5.2–9.6%]; *p* = 0.008) and 90 min (1.5% [1.5–1.8%] vs. 17.4% [14.9–20.2%]; *p* = 0.008) at room temperature (Figure 8A, Supplementary Figure S3). Adding acetaminophen (10 mg/mL) and NAPQI (125 µg/mL) to perfusate in vitro did not result in the development of significant methemoglobinemia (Figure 8B,C).

### 3.6. Carbon Tetrachloride (CCl_4_)

CCl_4_ was trialed as an alternative to acetaminophen in two perfused livers, but similar challenges were encountered. The first liver was treated with 10 mL of CCl_4_ and a second liver with 5 mL. The first was perfused for 1160 min and the second for 661 min. Both had higher peak ALT (518 and 298 U/L), AST (12,929 and 15,271 U/L), and LDH (8,912 and 8,486 U/L) compared to controls. End pH (6.99 and 7.13) and lactate (10.5 and 9.9 mmol/L) were similarly abnormal. Comparisons with controls were not statistically significant owing to the small sample size.

Hemolysis was particularly problematic in the CCl_4_-treated livers. Free hemoglobin was measured at 8.5 and 5.0 g/L by the end of perfusion. Hemoglobin concentration also decreased by 25 g/L in both perfusions. CCl_4_ as the causative agent of hemolysis was again demonstrated in vitro. CCl_4_ added to whole blood at a concentration of 10 µL/mL resulted in free hemoglobin of 13.67 g/L (5.41–15.48 g/L) after 30 min, compared to 0.37 g/L (0.30–0.38 g/L) in controls (*p* = 0.008) (Supplementary Figure S4A,B). In addition to the hemolysis, incompatibilities with circuit components (mainly the polycarbonate stopcocks and tubing connectors) made further attempts untenable (Supplementary Figure S4C).

## 4. Discussion

Our study set out to develop a large animal model of ALF in an isolated, perfused liver to abrogate ethical challenges, cost and complexity of toxicity studies in the intact pig. We were able to show that the addition of acetaminophen in the ex vivo setting resulted in massive liver injury. However, we found that the degree of injury was not well represented by perfusate biochemistry and was confounded by the presence of substantial methemoglobinemia and an overall increase in hemolysis. Additionally, we were able to demonstrate in vitro that the cause of the methemoglobinemia was due to PAP, as opposed to acetaminophen itself or its main hepatotoxic metabolite, NAPQI. We also performed precursory studies with CCl_4_ as an alternative hepatotoxic agent and identified major limitations with hemolysis for its use in the ex vivo setting.

Methemoglobinemia is a well described complication of acetaminophen toxicity in large animal models, including swine and canines [30,31]. However, it is only rarely seen in clinical cases of acetaminophen overdose [32]. This has made it challenging to find relevant preclinical models, as below a certain dose, animals tend to recover without developing lasting liver injury, while above it, they succumb to the effects of methemoglobinemia. Accumulation of methemoglobin differs from the mechanism by which acetaminophen is known to cause hepatocyte injury and fulminant liver failure, which results from an accumulation of NAPQI by cytochrome P450 metabolism of acetaminophen [33]. In contrast, PAP has been proposed as the metabolite responsible for methemoglobinemia and occurs as a result of deacetylation of acetaminophen [29]. Our in vitro studies confirmed that PAP readily induces methemoglobin formation in blood-base perfusate, whereas exposure to acetaminophen or NAPQI does not. The only swine models that have been able to overcome this challenge with methemoglobinemia have done so using titrated doses of acetaminophen over several hours [15,16].

Despite titrating acetaminophen dosing to methemoglobin level in the ex vivo setting, we were not able to avoid onset of experiment-limiting methemoglobinemia as the organ continued to be perfused. Methemoglobin develops as a result of the oxidation of the iron molecule in hemoglobin from the ferrous (Fe^2+^) to the ferric state (Fe^3+^) [34]. Whether porcine blood is more prone to methemoglobin formation, the additional cellular stress arising from exposure to the artificial components of the ex vivo circuit likely further contributed to its development [35]. Supporting this is the observation of methemoglobinemia developing after extended perfusion of discarded human livers [36].

Though the use of CCl_4_ in models of both acute and chronic liver injury has been well described, we found it unsuitable as an agent for hepatotoxicity in the ex vivo setting [37]. Doses were chosen based on a porcine model of ALF, in which CCl_4_ was injected directly into the portal vein [38]. However, this resulted in significant hemolysis, which both confounded the injury pattern and hampered perfusion of the liver in our model. CCl_4_ is typically dosed intraperitoneally or per os, which may mitigate its hemolytic effects in reported models [37]. We were unable to find mention of hemolysis amongst studies using CCl_4_ to induce liver failure. In addition, CCl_4_ proved to be quite a powerful solvent such that its mere infusion through polycarbonate stopcocks resulted in their degradation (Supplementary Figure S4). A corollary of this observation is that CCl_4_ may be useful for models of hemolytic anemia.

There are several alternative agents that could be considered to induce ALF. D-galactosamine has commonly been used, often in combination with lipopolysaccharide, to induce ALF in animal models [39]. As an amino sugar it is likely to be compatible with ex vivo perfusion components. However, it is not clear the degree to which immune-mediated damage is necessary to induce fulminant liver failure and whether that could be achieved in an isolated organ. In addition, it has been reported to have some batch-to-batch variability in potency, owing to is isolation from a biological source, which may affect model reproducibility depending on the dose required. Thioacetamide and azoxymethane are agents that have been used in vivo in rodent models [40,41]. However, there is limited experience with large animal models. As small molecule, biologically active chemicals, both would first warrant in vitro testing to ensure compatibility with porcine blood and circuit components. A-amanitin, the cyclic peptide produced by the *Amanta* genus of mushrooms, may be a more suitable candidate, as its structure is unlikely to cause unfavourable interactions with blood or artificial materials and, with its mechanism of RNA polymerase inhibition, it would be directly hepatotoxic [42]. There is even some experience with in vivo models in swine and non-human primates [43,44]. However, the cost of the molecule ($250–350 USD/mg) may be somewhat of a deterrent. Alternative measurements of liver injury that are more sensitive may be of use in developing consistency amongst such models, but ultimately they will need to show benefit by some clinically accepted parameter (such as transaminases or lactate clearance) to justify clinical translation.

Among important considerations for future use of isolated perfused organs as models of disease with the aim of achieving reproducibility are the development of standardized protocols for organ procurement, including the use of appropriate cold preservation solutions and cooling techniques if the organ is to be kept cold or minimizing warm ischemia if the organ is to be perfused immediately. As well, appropriate surgical skills are necessary to ensure organ procurement occurs efficiently, and the risks of undue injury are minimized. Similarly, protocols for organ perfusion itself, such perfusion pressures or flows and drug additives, should be consistent across the experiment and researchers should be comfortable with the technical aspects of their setup to allow for basic troubleshooting. If circuit components are to be reused (which is typical practice in the experimental setting), they should be cleaned thoroughly to avoid contamination between experiments and replaced entirely after a certain period of time or number of uses. Otherwise, routine experimental practices, such as maintaining standards for animal care and welfare, randomizing and blinding when possible, and using the same reagents between experiments, should be followed.

Efficient, reproducible large animal models of ALF are needed to develop effective treatments to screen for new interventional therapies that improve patient outcomes and reduce the need for liver transplantation. Ex vivo liver NMP offers a new platform on which to implement such models, with the additional benefit of reducing animal suffering and safely extending the duration of experiments. However, our early experience identified several challenging factors that hindered development of a reproducible model using two well-known hepatotoxic agents in this setting. Our finding from the present study provides useful insight to other investigators in the field, and description of this approach will provide additional information for researchers in the field of liver injury to improve their understanding of models of organ injury and aid the development of efficient animal models. Future studies altering the mode of delivery or trialing novel agents may be able to overcome these barriers to make use of NMP as a model for ALF.

## Data Availability

The data presented in this study are available in the article and supplementary material.

## Supplementary Materials

The following supporting information can be downloaded at: https://www.mdpi.com/article/10.3390/biomedicines10102496/s1, Supplementary Figure S1: Normothermic Machine Perfusion Schematic Diagram; Supplementary Figure S2: Perfusion Parameters; Supplementary Figure S3: Methemoglobinemia in vitro; Supplementary Figure S4: Carbon Tetrachloride in vitro.
