# Supplementary material for: Model of Acute Liver Failure in an Isolated Perfused Porcine Liver—Challenges and Lessons Learned
Source: Biomedicines. 2022 Oct 6;10(10):2496. doi: 10.3390/biomedicines10102496 (PMC9598959; doi:10.3390/biomedicines10102496)
Supplement: Supplementary file 1 [file biomedicines-10-02496-s001.zip › biomedicines-1860057-supplementary.pdf]

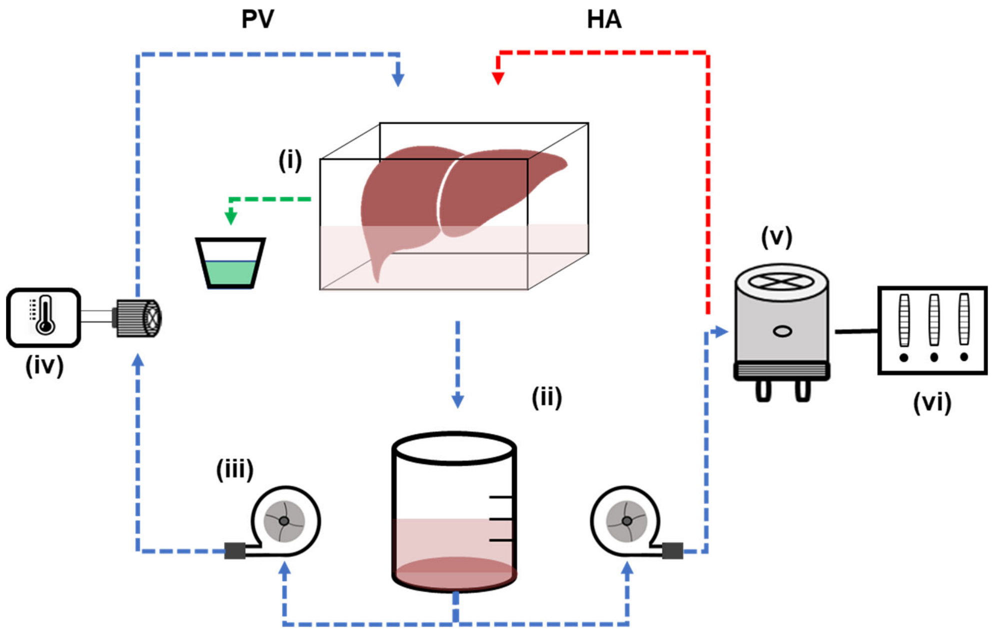

Supplementary Figure S1. Schematic of experimental ex vivo organ perfusion circuit. Perfusate drains from the organ chamber (i) into the reservoir (ii) and is recirculated by centrifugal pumps (iii). The pump on the left circulates perfusate through a heat exchanger (iv), while the pump on the right circulates perfusate through an oxygenator (v), which is supplied a physiologic gas mixture (vi). HA, hepatic artery; PV, portal vein.

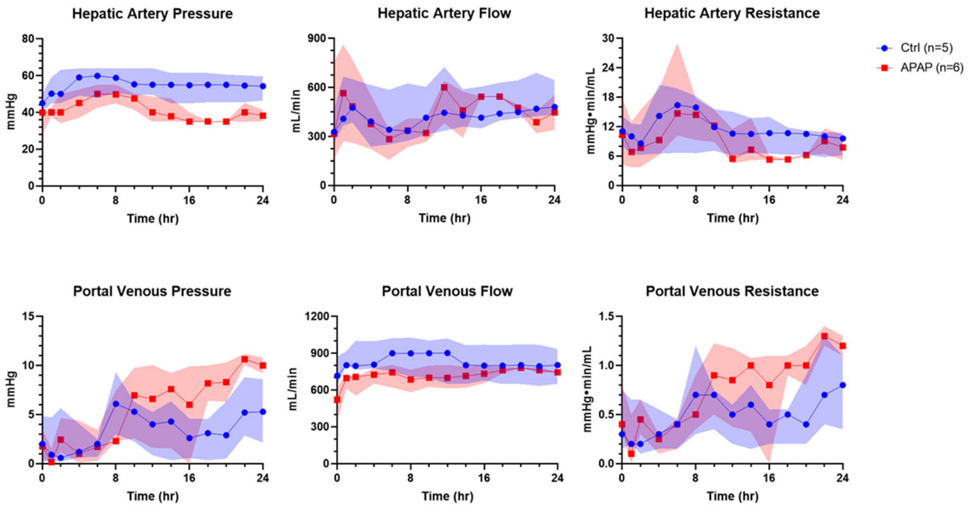

Supplementary Figure S2. Perfusion parameters for normothermic porcine liver perfusion, with (n-acetyl-para-aminophenol, APAP) or without (Ctrl) acetaminophen as a hepatotoxic agent. The highlighted area represents IQR.

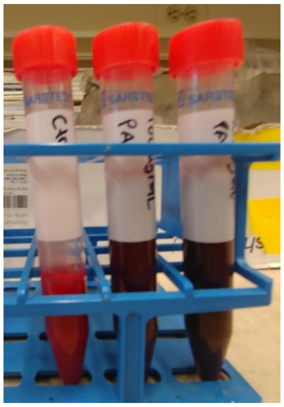

Supplemental Figure S3. Visualization of methemoglobinemia 90 minutes after the addition of p-aminophenol (PAP) to a sample of perfusate (1:1 Krebs-Henseleit and porcine whole blood). Tubes from left to right contain 0, 100, and 250μg/mL of PAP.

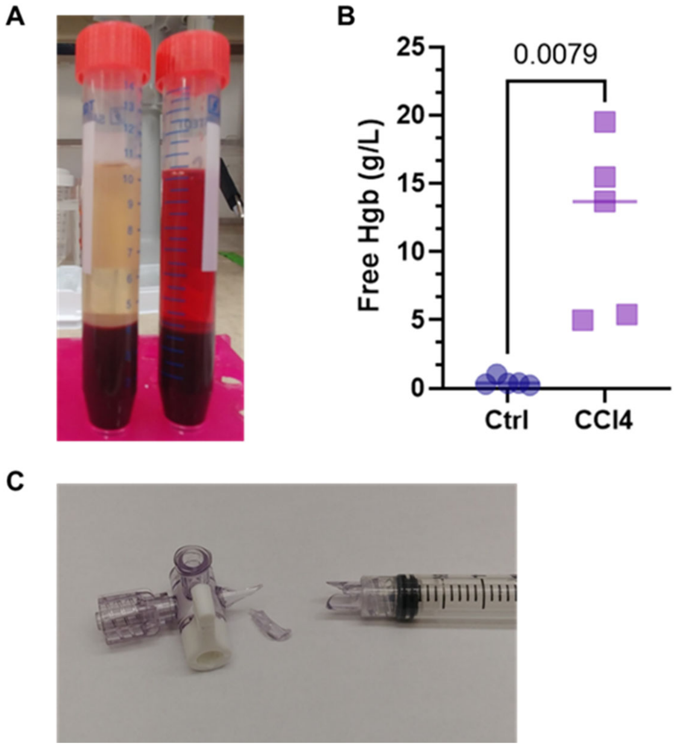

Supplementary Figure S4. Carbon tetrachloride (CCl_4) causes hemolysis *in vitro* in porcine whole blood samples, as demonstrated (**A**) visually and (**B**) by measurement of free hemoglobin after 30 minutes at room temperature. CCl_4 also degrades polycarbonate circuit components (**C**).
